# Novel mutations in the *HADHB* gene causing a mild phenotype of mitochondrial trifunctional protein (MTP) deficiency

**DOI:** 10.1002/jmd2.12276

**Published:** 2022-03-01

**Authors:** Kristin Ørstavik, Kjell Arne Arntzen, Per Mathisen, Paul Hoff Backe, Trine Tangeraas, Magnhild Rasmussen, Erle Kristensen, Marijke Van Ghelue, Christoffer Jonsrud, Yngve Thomas Bliksrud

**Affiliations:** ^1^ Department of Neurology, Section for Rare Neuromuscular disorders and EMAN Oslo University Hospital, Rikshospitalet Oslo Norway; ^2^ National Neuromuscular Centre Norway and Department of Neurology University Hospital of North Norway Tromsø Norway; ^3^ Department of Cardiology Oslo University Hospital, Rikshospitalet Oslo Norway; ^4^ Department of Microbiology Oslo University Hospital, Rikshospitalet and University of Oslo Oslo Norway; ^5^ Department of Medical Biochemistry Institute for Clinical Medicine, University of Oslo Oslo Norway; ^6^ Norwegian National Unit for Newborn Screening, Division of Pediatric and Adolescent Medicine Oslo University Hospital Oslo Norway; ^7^ Department of Clinical Neurosciences for Children Oslo University Hospital, Rikshospitalet Oslo Norway; ^8^ Department of Medical Biochemistry Oslo University Hospital, Rikshospitalet Oslo Norway; ^9^ Department of Medical Genetics, Division of Child and Adolescent Health University Hospital of North Norway Tromsø Norway

**Keywords:** HADHB, MTP, mutation, neuropathy, weakness

## Abstract

Mitochondrial trifunctional protein (MTP) deficiency is an ultrarare hereditary recessive disorder causing a broad spectrum of phenotypes with lethal infantile cardiomyopathy at the most severe end. Attenuated forms with polyneuropathy have been reported combined with myoglobinuria or rhabdomyolysis as key features. We here report three young adults (two siblings) in which three variants in the *HADHB*‐gene were identified. All three cases had a similar mild phenotype with axonal neuropathy and frequent intermittent weakness episodes but without myoglobinuria. Special dietary precautions were recommended to minimize complications especially during infections and other catabolic states. MTP deficiency is therefore an important differential diagnosis in patients with milder fluctuating neuromuscular symptoms.

**Take‐home message:**

Axonal neuropathy and recurrent muscular weakness without concomitant rhabdomyolysis may be due to MTP deficiency.

**Key Points**

‐ MTP deficiency usually gives rise to early and severe affection of several organs.

‐ Less severe phenotypes are reported but often including myopathy and rhabdomyolysis.

‐ In som cases a slight neuropathy with additional episodes of weakness without CK‐emia might be the only manifestation of this metabolic disorder.

‐ The effect of dietary treatment on these manifestations is so far unknown.

## 1 INTRODUCTION

Mitochondrial trifunctional protein (MTP) deficiency is a long‐chain fatty‐acid *β*‐oxidation disorder characterized by reduced activities of all three MTP enzymes. However, the most common defect of the MTP complex is isolated long‐chain L‐3‐hydroxyacyl‐CoA dehydrogenase (LCHAD) deficiency with considerable conservation of the other two MTP enzyme activities.^1^ Both MTP and isolated LCHAD deficiencies are rare autosomal recessive disorders caused by variants in the *HADHA* and *HADHB* genes coding for the α and β subunits of the trifunctional protein, respectively.^2^ The combined prevalence of MTP and LCHAD deficiency is estimated to be 1.02 per 100 000 live birth worldwide,^3^ and is in accordance with the findings in the Norwegian Newborn screening program (1:92000).^4^

MTP associated disorders can cause fatal cardiomyopathy, coma, and liver failure. The infants most severely affected may not be amenable to treatment and die within the first year of life.^4^, ^5^ Nevertheless, early dietary treatment might ameliorate the outcome of the disorders.^6^, ^7^ Additionally, less severe phenotypes including patients with polyneuropathy or myopathy are reported. The milder phenotypes are characterized by a combination of polyneuropathy, retinopathy, myopathy, and occasionally episodic rhabdomyolysis triggered by exercise or fever, infection, or reduced food intake.^8^ An MTP deficiency subtype with isolated axonal neuropathy resembling Charcot‐Marie‐Tooth type 2 (CMT2) has been described in a few cases from East Asia.^9^ A recent study also described neuropathy as the *only presenting* manifestation in four children with variants in the *HADHA* gene.^10^ In this article, we present three cases with an attenuated phenotype diagnosed in late adolescence/early adulthood without previous episodes of myoglobinuria, rhabdomyolysis, or raised creatine kinase (CK). All three patients have provided written informed consent.

## 2 CASE REPORTS

### 2.1 Case 1

Case 1 is a female patient, born to unrelated Norwegian parents. Uneventful course until 6 years of age when she started to gain extra weight and complained of decreased endurance during physical activity. At the age of 12, she was admitted to the University Hospital with pneumonia and developed generalized paresis. Her creatine kinase (CK) was below upper normal reference values (<250 U/L), and there was no discoloring of her urine. Electromyography (EMG) disclosed pathological spontaneous activity, whereas a muscle biopsy exhibited neurogenic findings. She regained the ability to walk independently 6 months after her hospital stay. At age 16, she was overweight (20 kg > 97.5 centile). Subsequently, weekly episodes of motor symptoms with weakness were reported, especially occurring in her lower limbs lasting minutes to hours, rendering her unable to walk. Neurological examination showed weak tendon reflexes, and she had difficulty walking on her heels. EMG showed a pattern typical for an axonal motor neuropathy, and she was diagnosed with a probable CMT2. In her 20s, she was in surgery for gastric bypass and had three uneventful pregnancies. As an adult, she had another episode of severe paresis lasting for weeks following gastroenteritis. However, her daily to weekly short‐lasting episodes of weakness persisted. Now in her early 30s, she shows progressive EMG and clinical findings compatible with sensorimotor polyneuropathy; decreased muscular strength in her feet with a tendency to pes cavus and thickened Achilles tendons, loss of biceps and Achilles tendon reflexes, and decreased sensibility for vibration and pin‐prick distally. CK and lactate have remained normal at all times. Genetic testing was performed using the IlluminaTruSight One Expanded Sequencing Panel (Illumina, San Diego, CA, USA), comprising probes to enrich 6704 genes prior sequencing. From this expanded panel, we selected a neuromuscular panel comprising 383 genes for further investigation (https://genetikkportalen.no/?act=genpan&katID=19&GpanID=21#popup108).

No variants typical for CMT2 were found. However, two variants in the *HADHB*‐gene (NM_000183.3) c.255‐1G > A p.(?) (SCV001244851.1) and c.998C > T (p.Pro333Leu) (SCV001244852.1)) were detected.

Parental segregation testing confirmed that she had inherited one variant from each of her parents. Targeted diagnostic metabolic screening revealed an increased concentration of long chain hydroxy‐acylcarnitine in a pattern compatible with long‐chain fatty acid oxidation disorder (Table 1). Enzyme activity measurements performed on lymphocytes (laboratory of Genetic Metabolic Diseases, Amsterdam UMC, the Netherlands [www.labgmd.nl]) revealed reduced long‐chain acyl‐CoA dehydrogenase (LCHAD) activity and markedly reduced long‐chain 3‐ketoacyl‐CoA thiolase activity (Table 1). The patient showed no cardiac, retinal, or liver involvement. She had no cognitive impairments, and brain magnetic resonance imaging (MRI) was normal. MRI of the muscles showed fat replacement especially in the proximal muscles of the lower limbs. She recently started a low‐fat diet supplemented with medium chain triglycerides (MCT) fat, but she still experiences episodes of weakness provoked by normal physical activity but also during nightly awakenings while resting in bed. After initiating fat restriction, the level of acylcarnitines has normalized.

**TABLE 1:** Biochemical and genetic results

Analyses (reference values)	♀ Case 1	♀ Case 2	♀ Case 3
C16:1‐OH‐carnitine μmol/L (0–0.017)	0.024	N	N
C18‐OH‐carnitine μmol/L (0–0.011)	0.013	N	N
C18:1‐OH‐carnitine μmol/L (0–0.013)	0.021	N	N
Long‐chain 3‐hydroxy acyl‐CoA dehydrogenase nmol/mg prot/min (22–74)	7 ↓↓	19 ↓	18 ↓
3‐Ketothiolase (long‐chain) nmol/mg prot/min (23–43)	3 ↓↓↓	15 ↓	13 ↓
Genetic analyses *HADHB*	c.255‐1G > A p.(?)	c.694G > A p.(Ala232Thr)	c.694G > A p.(Ala232Thr)
	c. 998C > T (p.Pro333Leu)	c.694G > A p.(Ala232Thr)	c.694G > A p.(Ala232Thr)

### 2.2 Case 2 and 3

Cases 2 and 3 are sisters in their 20s. They were born in a country in the western part of Asia and grew up in a refugee camp before arriving in Norway in their late teens. They both reported episodes of short‐lasting weakness (minutes) after moderate exercise and more long‐lasting weakness (days) in relation to febrile illnesses. No discoloring of urine or increased CK was reported. Clinical examinations between these episodes showed absent tendon reflexes in both sisters and a mild proximal weakness in the legs in the oldest sister. She was previously admitted to a University Hospital in Norway following a 4 weeks period of proximal muscle weakness starting after a viral respiratory infection. MRI showed contrast enhancement in lumbar meninges and nerve roots that could be consistent with Guillain‐Barré syndrome. However, her EMG showed a pattern of motor axonal involvement, and there was no increase in spinal protein. In addition, she had normal CK during this severe weakness episode. Muscle biopsy revealed neurogenic features. She received no treatment and recovered spontaneously.

Both sisters were homozygous for a mutation in the *HADHB*‐gene (NM_000183.3) c.694G > A p.(Ala232Thr).

Targeted metabolic screening during habitual state showed that both sisters had normal levels of long‐chain hydroxyl‐acylcarnitines. Free and total carnitine were also normal. On the other hand, enzyme activity was slightly diminished for both LCHAD and MTP (Table 1). There were no retinal findings and neither was obese. Echocardiography showed no cardiomyopathy, but a minor mitral valve insufficiency and a small mitral annular disjunction were present in one of the sisters. An increased right ventricle trabeculation was found in the other. Brain MRI in one of the sisters was normal. However, they both scored slightly below normal on cognitive tests.

## 3 DISCUSSION

In this report, we describe three adult patients with MTP deficiency with a similar phenotype of muscle weakness and/or polyneuropathy as their predominant disease manifestation but without concomitant or preceding episodes of CK elevations or rhabdomyolysis. The latter is the hallmark of long‐chain fatty‐acid‐oxidation (FAO) diseases, in particular during decompensations. Three variants including two novel ones were found in the *HADHB* gene and were originally classified as variants of uncertain significance (VUS) according to modified American College of Medical genetics and Genomics (ACMG) guidelines.^11^ None of these variants was seen in a homozygous state in the population database.

**FIGURE 1:**
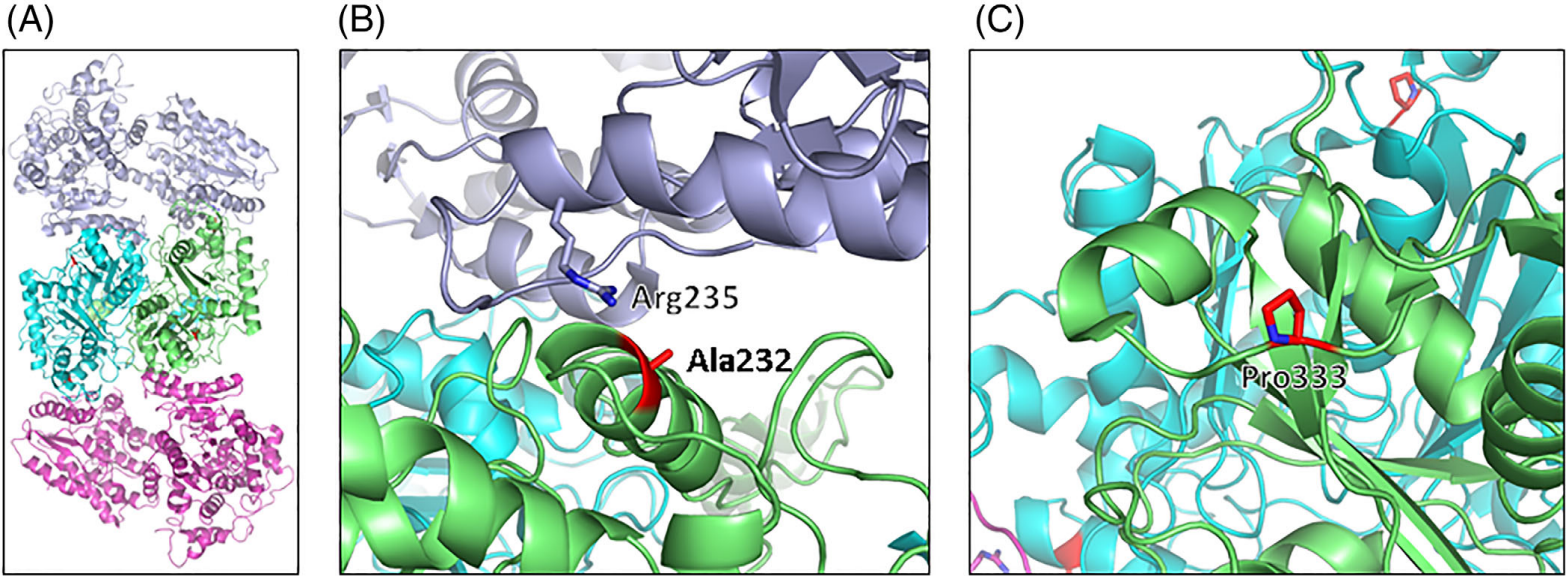
Cryo EM‐structure of the human mitochondrial trifunctional protein (MTP) complex.^14^ (A) MTPβ subunits form a homodimer located in the middle with one MTPα subunit on each side. The two MTPα subunits are shown in blue and magenta, and the two MTPβ subunits are shown in green and cyan. The amino acids MTPβ Ala232 and Pro333 are depicted in red. (B) Close‐up view of the Ala232 in MTPβ and the Arg235 in MTPα located in the interface between the α and β subunit. C) Close‐up view of MTPβ Pro333. The figures were prepared with PYMOL (http://www.pymol.org)

*HADHB* c.255‐1G > A is predicted to affect the acceptor splice site of intron 5. The consequences of such variants are unpredictable without experimental cDNA analysis, but exon 6 skipping is very likely to occur and may result in a frame shift with an early‐introduced stop codon. This variant is reported in a normal population database (GnomAD) with a low allele frequency (0.004%).^12^ The missense variant NM_000183.3 (*HADHB*):c.694G > A p.(Ala232Thr) found in case 2 and 3 is reported only twice among 251 342 alleles tested in the gnomAD (v2.1.1). Neither variant was reported in the clinical database Clinvar.^13^ Structural analysis was based on the recently published cryo‐electron microscopy α2β2 tetrameric structure of the human MTP.^14^ The structure shows that two TFPβ subunits form a homodimer that is located in the middle with one TFPα subunit on each side (Figure 1A).

Pro333 is located in a loop region in the MTPβ subunit (Figure 1B). Proline has more conformational rigidity compared to other amino acids and is highly conserved between species in this position. A substitution in this position might lead to reduced protein stability. Alignments were visualized in Alamut (Sophia Genetics Switzerland). Orthologs were taken from Emsembl Compara database (data are not shown).

The missense variant *HADHB* c.694G > A (Ala232Thr) found in case 2 and 3 is reported only once among 251 342 alleles tested in the GnomAD database, and a single entry was found in ClinVar where it was classified as a VUS (National Center for Biotechnology Information. ClinVar; [SCV000845642.1], https://www.ncbi.nlm.nih.gov/clinvar/variation/587640/ (accessed,Nov. 156 282 021)).

The hydrophobic Ala232 is placed in the interface between MTPα and MTPβ (Figure 1C). The substitution with the polar threonine might therefore destabilize the interaction between them. The previously reported MTPα Arg235Trp mutant strengthens the assumption that mutations within this interface region might be detrimental to MTP complex stability.^14^, ^15^, ^16^

Based on the clinical phenotypes and functional analysis (decreased residual enzyme activity), we assume that the three variants identified are likely pathogenic.

All patients experienced generalized mild muscular weakness and frequent short‐lasting episodes of attenuated decreased strength especially precipitated by physical activity and infections. Two patients (1 and 2) also had one to two serious and long‐lasting episodes of reversible paresis. Between weakness episodes, absent tendon reflexes and a mild proximal paresis in case 2 were present. However, case 1 exhibited clear signs of polyneuropathy. None of the patients had increased CK, rhabdomyolysis, or episodes of discolored urine. Electromyography findings were compatible with an axonal neuropathy in all cases. Muscle biopsies in case 1 and 2 were neurogenic.

A neuropathy resembling CMT2 was previously described in patients with variants in the *HADHB* gene. Lu and colleagues described two patients with axonal neuropathy, and one of them, an adult (19 years old), resembled our case 1.^17^ He had clinical and EMG findings compatible with an axonal neuropathy and no history of rhabdomyolysis or CK‐emia. However, he had no recurrent episodes of weakness. A pair of Korean adult siblings with *HADHB* variants were also diagnosed with CMT 2 and normal CK.^9^ However, they did not experience recurrent episodes of weakness either. Recently, Nadjar and colleagues described four MTP cases with a new phenotype with adult‐onset ataxia and sensory ganglionopathy, but these were preceded by rhabdomyolysis in childhood in all cases.^18^ A newly published study on patients with MTP deficiency and neuropathy describes neuropathy as the only presenting symptom in four children.^10^ However, variants in the *HADHA* gene were the likely cause of the metabolic disorder in these cases. On the other hand, in the cases presented in the latter study, the long‐lasting episodes of weakness were reversible, mimicking the findings in our three adult patients.

Several authors have described episodes of weakness after vigorous exercise in relation to MTP deficiency. These episodes are often named exercise‐induced myopathy.^2^, ^8^, ^19^ However, these episodes are vaguely characterized and often coupled to some degree of increased CK. The mechanism for these episodes is probably muscle exhaustion since long‐chain fatty acids are the predominant fuel in the skeletal muscle. In case 1, the patient also experienced episodes of weakness during the night when she was unable to stand up; this might be coupled to a state of catabolism.

The two sisters had a mild cognitive impairment. This could be related to the slight MTP deficiency but must be interpreted with caution, as medical records were not available from early childhood. Apart from this, no major organ involvement was found in any of the patients until now. Opthalmological examinations and cardiac function are followed on a regular basis.

Our three cases instituted the recommended diet treatment for long‐chain FAO only in late adolescence including a long‐chain‐fat‐restricted diet, MCT supplementation, fasting restrictions, and glucose polymer emergency regime during intercurrent illness. However, as a recent review showed, the long‐term benefits of these strict dietary regimes are poorly studied, and further studies are needed to evaluate the impact of diet on attenuated phenotypes.^7^ In Norway, newborn screening (NBS) for fatty acid oxidation disorders (including LCHAD/TFP) was implemented in 2012,^4^ and in Europe, LCHAD/TFP are currently part of the core NBS conditions in about 20 countries.^20^ Early detection by NBS has been shown to improve mortality and morbidity for FAO conditions as a group.^21^ A systematic review regarding treatment of early‐ versus late‐diagnosed LCHAD/TFP patients showed better hepatic and cardiac status.^7^ However, other complications such as neuropathy and retinopathy failed to show any difference in outcome. The authors concluded that the individual studies had small sample size, and that a call for collaborative research studies were needed to decipher benefit from early treatment by NBS.^7^

In conclusion, MTP deficiency should be included in the differential diagnosis even in young adult patients with unknown etiology of an axonal neuropathy and especially if there are fluctuating motor symptoms in relation to exercise and infections. Normal acylcarnitines, lack of elevated CK, and no history of rhabdomyolysis do not exclude a diagnosis of MTP deficiency.

## ETHICAL APPROVAL

Kristin Ørstavik, Kjell Arne Arntzen, Per Mathisen, Paul Hoff Backe, Trine Tangeraas, Magnhild Rasmussen, Erle Kristensen, Marijke Van Ghelue, Christoffer Jonsrud, and Yngve Thomas Bliksrud have nothing to disclose. All procedures were in accordance with the ethical standards of the responsible committee on human experimentation (institutional and national) and with the Helsinki Declaration of 1975, as revised in 2000 (5). Informed consent was obtained from all 3 patients for being included in the study. The publication of the case report was approved by the Institutional Data Protection Officer.

## Data Availability

More clinical data are available on request. None of the authors had any conflicts of interest.
